# Changes in Biomass, Mineral Composition, and Quality of Cardoon in Response to ${\mathrm{NO}}_{3}^{-}$:Cl^-^ Ratio and Nitrate Deprivation from the Nutrient Solution

**DOI:** 10.3389/fpls.2016.00978

**Published:** 2016-06-30

**Authors:** Daniela Borgognone, Youssef Rouphael, Mariateresa Cardarelli, Luigi Lucini, Giuseppe Colla

**Affiliations:** ^1^Department of Agricultural and Forestry Sciences, Tuscia University, ViterboItaly; ^2^Department of Agricultural Sciences, University of Naples Federico II, PorticiItaly; ^3^Consiglio per la Ricerca in Agricoltura e l’Analisi dell’Economia Agraria, Centro di Ricerca per lo Studio delle Relazioni tra Pianta e Suolo, RomaItaly; ^4^Institute of Environmental and Agricultural Chemistry, Università Cattolica del Sacro Cuore, PiacenzaItaly

**Keywords:** antioxidant capacity, *Cynara cardunculus* L. var. *altilis* DC, nitrate/chloride ratio, secondary metabolism, floating system, total phenols

## Abstract

Leaf extracts of cultivated cardoon (*Cynara cardunculus* L. var. *altilis* DC) are an important source of phenols. Soilless culture represents an important and alternative tool to traditional agriculture, since it allows a precise control of plant nutrition and the maximization of yield and quality of the product. Reducing N supply, while keeping quantity as high as possible is desirable for environmental and health-related reasons, especially that N deficiency can lead to improved concentrations of secondary plant metabolites. Two greenhouse experiments were carried out in order to determine the effect of a decreasing ${\mathrm{NO}}_{3}^{-}$:Cl^-^ ratio (80:20, 60:40, 40:60, or 20:80) and nitrate deprivation (0, 5, 10, or 15 days before harvest) on biomass production, leaf chlorophyll content and fluorescence, mineral composition, and phytochemicals in leaves of cardoon ‘Bianco Avorio’ grown in a floating system. Total phenols, flavonoids and antioxidant capacity increased linearly with Cl^-^ availability, especially when nitrate was replaced by 80% of chloride (20:80 ${\mathrm{NO}}_{3}^{-}$:Cl^-^ ratio), without having a detrimental effect on yield. Total nitrogen and nitrate concentration in leaves decreased linearly with increasing Cl^-^ in the nutrient solution. Total phenols and antioxidant capacity recorded after 15 days of nitrate deprivation were higher by 43.1, 42.8, and 44.3% and by 70.5, 40.9, and 62.2%, at 59, 97 and 124 days after sowing, respectively compared to the control treatment. The decrease in leaf nitrate content recorded under N-deprivation occurred more rapidly than the reduction in total nitrogen. Thus, up to 15 days of nitrate withdrawal can lower nitrates without sharply reduce total nitrogen or affecting growth and biomass of cultivated cardoon. The use of N-free nutrient solution prior to harvest or the replacement of nitrates with chlorides could be adopted among growers to improve the quality of the product and enhance sustainability of crop production system.

## Introduction

*Cynara cardunculus*, a Mediterranean perennial species within the *Asteraceae* (*Compositaeae*) family, includes the two cultivated taxa globe artichoke (var. *scolymus*) and cardoon (var. *altilis* DC), along with their progenitor the wild cardoon also called artichoke thistle [var. *sylvestris* (Lamk) Fiori] (Rottenberg and Zohary, 1996; Portis et al., 2005; Calabrese et al., 2012). The cultivation of cardoon is less diffused than that of globe artichoke; its production is located in the Mediterranean areas of southern Europe (e.g., Italy, France, Greece, Portugal, and Spain; Portis et al., 2005).

Traditionally, cardoon is cultivated for the fleshy leaf petiole and part of the central leaf vein used to prepare typical Italian dishes (Cravero et al., 2012). In addition to the described traditional uses, this botanical variety has been also considered as ‘multipurpose perennial crop’ for several industrial applications due to its high biomass productivity (29.3 t ha^-1^; Ierna et al., 2012) and high dry matter content (Cravero et al., 2012). Therefore, in the last years this plant has been assessed as ‘biomass crop’ for paper pulp production, domestic heating, and power generation (Fernández et al., 2006; Ciancolini et al., 2013). Besides the high content of cellulose and hemicelluloses cultivated cardoon can be further valorized throughout the utilization of high-value extractable chemical components of the leaves. *Cynara* leaf extracts have been used since ancient times in folk medicine, due to their therapeutic potential as choleretic, hepatoprotective, antidiabetic and anticholestatics activities (Gebhardt and Beck, 1996; Gebhardt, 1997). These physiological properties have been mostly attributed to the content of phenylpropanoids (flavonoids, mono-, and di-caffeoylquinic acids) and sesquiterpene lactones (Lattanzio et al., 2009; Menin et al., 2010; Pandino et al., 2015).

Cardoon for leaf extract production is traditionally cultivated in Italy under open-field conditions with high-density plantation. Despite the relatively high interest of growers and pharmaceutical industries in cardoon leaf extracts, advanced cultivation techniques such as floating system have received little attention so far (Rouphael et al., 2012a; Colla et al., 2013a). Floating system is the most diffused cultivation soilless system for the production of fresh-cut leafy vegetables (Tomasi et al., 2015). Compared with traditional soil-based culture, floating system offers the possibility (i) to shorten the growing cycle with year round production, (ii) to reduce manpower cost, (iii) to avoid soil limitations (i.e., soil-borne pathogens, salinity, pollution), (iv) to grow plants at high density, (v) as well as to make more efficient the use of water and fertilizers (Fallovo et al., 2009a,b; Tomasi et al., 2015). Moreover, manipulation of target molecules such as antioxidants and phytochemicals by management of nutrient solution is a research area attracting interest of many scientists (Fanasca et al., 2006a,b). Nutrient solution recipes used in hydroponics have been usually formulated with high concentration of nitrate-the main N source for most crops, to reach maximal yield (Gruda, 2009). Minimizing N supply, while keeping quantity and especially quality as high as possible represents a major sustainability challenge, since many growers are faced with environmental and economical concerns (Colla et al., 2010, 2011). Recently, many investigations have shown that low N availability increases for example the concentrations of phenolics and flavonoids in several leafy vegetables (Stefanelli et al., 2010; Becker et al., 2015). In this perspective, floating system represents an effective way since it permits the precise control of plant nutrition (Rouphael et al., 2012a; Colla et al., 2013a; Borgognone et al., 2014). For instance, nitrate concentration in the nutrient solution can be reduced by replacing nitrate with chloride or by eliminating it several days before harvest (DBH). To our knowledge, no published data is available concerning the effects of ${\mathrm{NO}}_{3}^{-}$:Cl^-^ ratio and nitrate deprivation before harvesting on cardoon yield and quality. Our hypothesis is that a controlled nitrogen limitation in the nutrient solution by nitrate withdrawal or replacement with chloride may raise the quality of cardoon while maintaining yield.

To verify the current hypothesis, two greenhouse experiments were carried out in order to assess the effect of a decreasing ${\mathrm{NO}}_{3}^{-}$:Cl^-^ ratio (Experiment 1) and nitrate deprivation (Experiment 2) on biomass production, leaf chlorophyll content and fluorescence, mineral composition, total phenols, flavonoids and antioxidant activity in leaves of cardoon grown in a floating system.

## Materials and Methods

### Growth Conditions, Treatments, and Nutrient Solutions

Two experiments were conducted, one in 2013 (${\mathrm{NO}}_{3}^{-}$:Cl^-^ ratio experiment; Experiment 1) and the other in 2013–2014 (time course N deprivation experiment; Experiment 2), in a 300 m ×300 m polyethylene greenhouse situated at the experimental farm of Tuscia University, central Italy (latitude 42° 25′ N, longitude 12° 08′, altitude 310 m). The greenhouse was maintained at daily temperature between 10 and 30°C and day/night relative humidity 55/80%.

In both experiments, seeds of *Cynara cardunculus* L. var. *altilis* cv. ‘Bianco Avorio’ (La Semiorto Sementi, Lavorate di Sarno, Italy) were sown in polystyrene plug trays on 27 August 2013 (Experiment 1) and 7 December 2013 (Experiment 2). ‘Bianco Avorio’ was selected due to its high productivity, leaf quality and the adaptability to grow in floating raft culture (Rouphael et al., 2012a; Colla et al., 2013a). At two-true-leaf stage [23 and 21 days after sowing (DAS) in Experiments 1 and 2, respectively] cardoon plants were moved to a floating raft growing system. The floating raft system consisted of the polystyrene plug trays floating in plastic tanks with a constant volume of 60 L of aerated nutrient solution. An air compressor maintained the dissolved oxygen content above 6 mg L^-1^. The plant density was 463 plants m^-2^, as used commercially for baby leaf vegetables in floating system.

A randomized complete-block design with four replicates was used to compare four different ratios of ${\mathrm{NO}}_{3}^{-}$ to Cl^-^ (80:20, 60:40, 40:60, or 20:80) in Experiment 1 and four time course nitrate deprivation (0, 5, 10, or 15 DBH) in Experiment 2. Each experimental unit consisted of polystyrene plug trays (0.1815 m^2^) containing 84 plants. In Experiments 1 and 2, the composition of the basic nutrient solution was: 1.5 mmol L^-1^ P, 4.5 mmol L^-1^ K, 6.5 mmol L^-1^ Ca, 2 mmol L^-1^ Mg, 20 μmol L^-1^ Fe, 9 μmol L^-1^ Mn, 0.3 μmol L^-1^ Cu, 1.6 μmol L^-1^ Zn, 20 μmol L^-1^ B, and 0.3 μmol L^-1^ Mo. In Experiment 1, the four ${\mathrm{NO}}_{3}^{-}$ to Cl^-^ ratios were obtained by adding to the basic nutrient solution different amounts of calcium nitrate and calcium chloride (**Table 1**). In Experiment 2, nitrates (10 mmol L^-1^ NO_3_) was removed totally from the nutrient solution at 0, 5, 10, or 15 DBH, and calcium nitrate (15.5% of N) was replaced by calcium chloride (analytical grade) in order to keep constant Ca concentration. In experiments, the electrical conductivity and pH of the nutrient solutions in all treatments were 2.0 ± 0.2 dS m^-1^ and 6.0 ± 0.3, respectively. To prevent large fluctuation in the anions/cations concentrations, electrical conductivity and pH, the nutrient solution in all treatments were renewed from all tanks weekly.

**Table 1:** Nitrate concentration and salt quantity in the nutrient solution in the four different ${\mathrm{NO}}_{3}^{-}$:Cl^-^ ratios tested in cardoon (*Cynara cardunculus* L. var. *altilis* DC) grown in a floating system under greenhouse conditions (Experiment 1).

Treatment ${\mathrm{NO}}_{3}^{-}$:Cl^-^	${\mathrm{NO}}_{3}^{-}$ mM	Ca(NO_3_)_2_ mg l^-1^	CaCl_2_ mg l^-1^
80: 20	9.6	867.1	133.2
60: 40	7.2	650.3	266.4
40: 60	4.8	433.5	399.6
20: 80	2.4	216.8	532.8

### Leaf Biomass Determination

In both experiments, the leaves of cultivated cardoon were mowed three times during the growing cycles (45, 73, and 101 DAS in Experiment 1 and 59, 97, and 124 DAS in Experiment 2) when the plant height reached 25 cm. At each harvest date, the leaf tissues were dried in a forced-air oven at 60°C for 72 h for dry biomass determination. In both experiments, the dried leaf tissue of each harvest was used for mineral and quality analysis.

### SPAD Index and Fluorescence Measurements

In experiments, the Soil Plant Analysis Development (SPAD) index and the fluorescence measurements were recorded at each harvest time. A portable chlorophyll meter (SPAD-502, Minolta corporation, Ltd., Osaka, Japan) was used to measure the relative leaf chlorophyll concentration as a rational unit. Measurements were made at the central point of the leaflet between the midrib and the leaf margin of the third leaf starting from the apical shoot. Twenty random readings per plot were taken and averaged to a single SPAD value for each treatment

On the same dates, the chlorophyll fluorescence was recorded on 20 min dark-adapted leaves (in 3rd – 4th from top; three measurements per plant) by mean of a chlorophyll fluorometer Handy PEA (Hansatech Instruments Ltd, King’s Lynn, UK) with an excitation source intensity higher than 3000 μmol m^-2^ s^-1^ at the sample surface. The minimal fluorescence intensity (F_0_) in a dark-adapted state was measured in the presence of a background weak light signal (about 2–3 μmol photons m^-2^ s^-1^). The maximal fluorescence level in the dark-adapted state (F_m_) was induced by 0.8 s saturating light pulse (3000 μmol photons m^-2^ s^-1^). The maximum quantum yield of open photosystem II (PSII) (F_v_/F_m_) was calculated as (F_m_ – F_0_)/F_m_, as described by Arena et al. (2005).

### Mineral Analysis

In both experiments, the dried leaf tissues of cultivated cardoon, harvested three times during the growing cycles, were ground in a Wiley mill to pass through a 20-mesh screen, then 1 g samples were analyzed for nitrogen and nitrate, and also for chloride but only in Experiment 1. Nitrogen concentration in leaf tissues was determined after mineralization with sulfuric acid in the presence of potassium sulfate and a low concentration of copper by the Kjeldahl method (Bremner, 1965). Nitrate concentration in dry leaf was determined using the salicylic-sulfuric acids method (Cataldo et al., 1975) by spectrophotometry (Helios Beta Spechtophotometer, Thermo Electron Corporation, UK). Chloride ion concentration was determined by dry ashing at 400°C for 24 h, dissolving the ash in nitric acid (HNO_3_, 1:20 w/v), and assaying the solution by titration with AgNO_3_ in the presence of K_2_CrO_4_ (Eaton et al., 1995).

### Analysis of Total Phenolics and Flavonoids

Dried cardoon leaf sample (100 mg) was extracted sequentially three times in 10, 5, and 5 mL of fresh ethanol/water (80:20 v/v) containing 1 mL L^-1^ of 370 g L^-1^ HCl (total volume 25 mL). The extraction mixture was stirred for 10 min and then solids were removed by centrifugation at 503 × g for 5 min. The total content of phenolic compounds in each extract was determined by the Folin–Ciocalteu method (Singleton and Rossi, 1965). A calibration curve was prepared using aliquots of gallic acid ethanolic solutions, and results were expressed as gallic acid equivalents (mg gallic acid g^-1^ dry weight). All reagents and solvents used to prepare standard solutions and to extract phenols were of analytical grade and were purchased from Sigma Aldrich (Milan, Italy).

Total flavonoids were determined following the colorimetric aluminum chloride method reported by Dutta and Maharia (2012). A calibration curve was prepared using aliquots of quercitin (Sigma Aldrich, Milan, Italy), and results were expressed as quercetin equivalents (mg quercetin g^-1^ dry weight).

### Antioxidant Activity Determination

Antioxidant activity was determined by the ferric reducing-antioxidant power (FRAP) following the method described by Pellegrini et al. (2003). A calibration curve was prepared with increasing concentrations of FeSO_4_ and results were expressed as μmol FeSO_4_ g^-1^ dry weight. All reagents were of analytical grade and were purchased from Sigma Aldrich (Milan, Italy).

### Statistical Analysis

Analysis of variance (ANOVA) of the experimental data was calculated using the software package, SPSS 10 for Windows, 2001 (SPSS Inc., Chicago, IL, USA). In both experiments, orthogonal linear and quadratic polynomial contrasts were used to compare the different ratios of ${\mathrm{NO}}_{3}^{-}$ to Cl^-^ and the nitrate deprivation effects at different DBH on selected parameters (Gomez and Gomez, 1983).

## Results

### ${\mathrm{NO}}_{3}^{-}$:Cl^-^ Ratio Experiment

In Experiment 1, no significant difference in leaf dry biomass was observed between treatments when harvested at different DAS (data not shown). The mean leaf dry biomass of cardoon plants was 0.80, 0.63, and 0.42 g plant^-1^ at 45, 73, and 101 DAS respectively, whereas the total biomass of cardoon recorded at the end of the experiment was 1.86 g plant^-1^ DW (data not shown).

Similarly to dry biomass, the SPAD index and the maximum quantum use efficiency of the PSII in dark-adapted state measured as F_v_/F_m_ ratio were not affected by the ${\mathrm{NO}}_{3}^{-}$:Cl^-^ ratio. The mean SPAD index in leaves was 29.9, 31.4, and 26.6 at 45, 73, and 101 DAS, respectively, while the mean F_v_/F_m_ ratio in cardoon leaves was 0.84, 0.82 and 0.84, at 45, 73, and 101 DAS, respectively (data not shown).

The nitrogen (N), nitrate and chloride concentrations of cardoon ‘Bianco Avorio’ were significantly affected by the ${\mathrm{NO}}_{3}^{-}$:Cl^-^ ratio (**Table 2**). At 73 and 101 DAS, the N and nitrate concentration in leaves decreased linearly with increasing Cl^-^ in the nutrient solution. The chloride concentration increased linearly with the increase of Cl^-^ in the nutrient solution (**Table 2**).

**Table 2:** Effect of ${\mathrm{NO}}_{3}^{-}$:Cl^-^ ratio on total nitrogen (N), nitrate (${\mathrm{NO}}_{3}^{-}$), and chloride **(Cl^-^)** contents in leaves of cardoon plants (*Cynara cardunculus* L. var. *altilis* DC) harvested at different days after sowing (DAS); cardoon plants were grown in a floating system under greenhouse conditions (Experiment 1).

Treatment	N (g kg^-1^ DW)			${\mathrm{NO}}_{3}^{-}$ (g kg^-1^ DW)			Cl^-^ (g kg^-1^ DW)		
${\mathrm{NO}}_{3}^{-}$:Cl^-^	45 DAS	73 DAS	101 DAS	45 DAS	73 DAS	101 DAS	45 DAS	73 DAS	101 DAS
80: 20	42.48	41.50	46.91	16.78	16.63	10.53	29.03	27.97	27.23
60: 40	44.32	40.30	43.91	12.70	14.36	8.67	31.72	35.02	33.38
40: 60	41.89	36.76	42.23	10.36	10.67	7.01	38.07	41.10	35.09
20: 80	41.53	35.96	37.11	8.82	7.56	7.66	48.97	40.49	38.83
Significance	NS	L**	L***	L**	L***	L*	L***	L**	L***

L, linear; Q, quadratic; Ns, non-significant; *P < 0.05; **P < 0.01; ***P < 0.001.

Total phenolics (TP), total flavonoids (TF), and antioxidant activity (FRAP) were significantly affected by the ${\mathrm{NO}}_{3}^{-}$:Cl^-^ ratio, except for TF and FRAP at 101 DAS, where no significant effect was observed between treatments (**Table 3**). Cardoon leaf extract quality (i.e., TP, TF, and FRAP) increased linearly with Cl^-^ availability, especially when nitrate was replaced by 80% of chloride (20:80; **Table 3**). However, for chloride concentration between 40 and 60%, the TP, TF, and FRAP remained unchanged. Finally, irrespective of the ${\mathrm{NO}}_{3}^{-}$:Cl^-^ ratio, the highest values of TP, TF, and FRAP were recorded at 101 DAS (**Table 3**).

**Table 3:** Effect of ${\mathrm{NO}}_{3}^{-}$:Cl^-^ ratio on total phenols, total flavonoids and ferric-reducing antioxidant power (FRAP) in leaves of cardoon plants (*Cynara cardunculus* L. var. *altilis* DC) harvested at different days after sowing (DAS); cardoon plants were grown in a floating system under greenhouse conditions (Experiment 1).

Treatment	Total phenols (mg gallic acid g^-1^ DW)			Total flavonoids (mg quercitin g^-1^ DW)			FRAP (μmol FeSO_4_ g^-1^ DW)		
${\mathrm{NO}}_{3}^{-}$:Cl^-^	45 DAS	73 DAS	101 DAS	45 DAS	73 DAS	101 DAS	45 DAS	73 DAS	101 DAS
80: 20	7.63	13.97	17.22	3.27	21.36	38.16	92.52	217.20	290.97
60: 40	9.03	15.04	19.34	6.97	24.69	45.89	117.11	247.37	318.04
40: 60	9.51	16.35	19.74	7.26	29.94	44.79	125.46	283.88	322.17
20: 80	10.59	22.09	21.03	8.82	49.55	54.62	144.65	390.45	371.98
Significance	L*	L**	L*	L*	L*	NS	L*	L***	NS

L, linear; NS, non-significant; *P < 0.05; **P < 0.01; ***P < 0.001.

### Nitrate Deprivation Experiment

In Experiment 2, the dry mass of cardoon leaves and total biomass values were not significantly affected by nitrate deprivation from the nutrient solution (data not shown). The average leaf biomass production was 0.40, 0.82, and 0.46 g plant^-1^ DW at 59, 97 and 124 DAS, respectively, whereas the mean total leaf dry biomass was 3.09 g plant^-1^ (data not shown).

At the last harvest, 124 DAS, the SPAD index decreased linearly with the increase in the number of days of nitrate deprivation (**Table 4**). Moreover, there were no statistical differences between treatments in the fluorescence measurements (**Table 4**).

**Table 4:** Effect of nitrate deprivation at different days before harvest (DBH) on SPAD index and maximum quantum use efficiency of PSII (F_v_/F_m_) in dark-adapted state of cardoon plants (*Cynara cardunculus* L. var. *altilis* DC) harvested at different days after sowing (DAS); cardoon plants were grown in a floating system under greenhouse conditions (Experiment 2).

Nitrate deprivation	SPAD index			F_v_/F_m_		
DBH	59 DAS	97 DAS	124 DAS	59 DAS	97 DAS	124 DAS
0	28.5	32.6	34.7	0.80	0.84	0.83
5	25.4	34.2	33.5	0.81	0.84	0.83
10	27.1	36.5	30.8	0.81	0.84	0.83
15	25.4	33.7	28.0	0.77	0.84	0.83
Significance	NS	NS	L***	NS	NS	NS

L, linear; NS, non-significant; ***P < 0.001.

The N concentration in leaf tissue decreased linearly at 59 and 124 DAS and quadratically at 97 DAS, with increase in number of days of nitrate deprivation from the nutrient solution (**Table 5**). Similarly, the nitrate concentration in leaves of cardoon plants, harvested at different DAS decreased linearly with nitrate withdrawal from the nutrient solution with a sharp reduction (83–87%) observed after 15 days of nitrate deprivation in comparison to control treatment (**Table 5**).

**Table 5:** Effect of nitrate deprivation at different days before harvest (DBH) on nitrogen (N), and nitrate (${\mathrm{NO}}_{3}^{-}$), contents in leaves of cardoon plants (*Cynara cardunculus* L. var. *altilis* DC) harvested at different days after sowing (DAS); cardoon plants were grown in a floating system under greenhouse conditions (Experiment 2).

Nitrate deprivation	N (g kg^-1^ DW)			${\mathrm{NO}}_{3}^{-}$ (g kg^-1^ DW)		
DBH	59 DAS	97 DAS	124 DAS	59 DAS	97 DAS	124 DAS
0	49.17	38.56	43.79	19.87	10.91	8.89
5	41.54	42.17	34.96	17.83	5.63	5.76
10	41.49	43.73	35.83	14.53	3.91	4.66
15	39.00	29.34	31.56	11.74	1.42	1.52
Significance	L*	Q*	L**	L***	L***	L***

L, linear; Q, quadratic; Ns, non-significant; *P < 0.05; **P < 0.01; ***P < 0.001.

The bioactive compounds (i.e., TP and TF) and antioxidant activity of cardoon leaf extract harvested at different DAS increased linearly, with the highest values recorded in the 15 days of nitrate deprivation treatment (**Table 6**). Finally, irrespective to nitrate withdrawal from the nutrient solution, the highest values of TP, TF, and FRAP were recorded at 97 DAS (**Table 6**).

**Table 6:** Effect of nitrate deprivation at different days before harvest (DBH) on total phenols, total flavonoids and ferric-reducing antioxidant power (FRAP) in leaves of cardoon plants (*Cynara cardunculus* L. var. *altilis* DC) harvested at different days after sowing (DAS); cardoon plants were grown in a floating system under greenhouse conditions (Experiment 2).

Nitrate deprivation	Total phenols (mg gallic acid g^-1^ DW)			Total flavonoids (mg quercitin g^-1^ DW)			FRAP (μmol FeSO_4_ g^-1^ DW)		
DBH	59 DAS	97 DAS	124 DAS	59 DAS	97 DAS	124 DAS	59 DAS	97 DAS	124 DAS
0	10.00	23.18	15.23	10.67	38.55	12.21	130.11	401.82	194.05
5	11.27	20.34	17.11	14.80	25.34	18.08	157.58	343.02	231.51
10	11.10	25.03	20.09	14.42	42.20	30.30	151.25	438.93	294.07
15	14.31	33.12	21.99	25.21	75.51	31.85	221.81	566.17	314.74
Significance	L*	L*	L***	L**	L*	L***	L*	L*	L***

L, linear; *P < 0.05; **P < 0.01; ***P < 0.001.

## Discussion

Management of the growing conditions, in particular the nutrient solution composition, is one of the most important aspect for successful leafy crop production in floating raft culture (Fallovo et al., 2009b; Rouphael et al., 2012a). In the current experiments, we have illustrated that minimizing the nitrate supply either by partial substitution of nitrate with chloride, or by nitrate deprivation several DBH, could be proposed as an effective tool to raise the quality of the cardoon leaves while maintaining yield.

It is well established that biomass production and marketable yield decrease with increasing salinity, as it has been demonstrated in many sensitive to moderately salt-tolerant greenhouse vegetables such as cucumber, melon, pepper, watermelon and zucchini squash grown hydroponically (Colla et al., 2006a,b,c, 2012, 2013b; Rouphael et al., 2006, 2012c). Ions, mainly Na^+^ and/or Cl^-^ in high concentration generates oxidative stress in plant tissues as revealed by the production of reactive oxygen species (ROS; Sreenivasulu et al., 2000) leading to several morphological, physiological and biochemical changes (Grattan and Grieve, 1999). High levels of Na^+^ and/or Cl^-^ in the root zone decreased the net nitrate uptake rate and nitrate translocation to the shoot in many vascular plants. This was the case in the current study (Experiment 1), since replacing nitrate by 80% of chloride (20:80) decreased nitrates in cardoon leaves as demonstrated in previous studies on leafy vegetables grown hydroponically (Urrestarazu et al., 1998; Neocleous et al., 2014).

Touraine et al. (1994) reported that salinity stress can reduce the growth rates of the plants, leading to an indirect downregulation of net nitrate uptake rate via a lower internal demand for N. This was not the case of Experiment 1 since shoot dry biomass was unaffected to any level of chloride in the nutrient solution. Rubinigg et al. (2003) found that root to shoot translocation of N was severely inhibited relative to the net nitrate uptake ratio at all NaCl concentrations applied. Anion channels with similar permeability for both ${\mathrm{NO}}_{3}^{-}$ and Cl^-^ have been found in xylem parenchyma cells from barley (Köhler and Raschke, 2000). These channels were suggested to play an important role in the xylem loading of ${\mathrm{NO}}_{3}^{-}$ and Cl^-^. In the presence of high Cl^-^ levels, root to shoot translocation of ${\mathrm{NO}}_{3}^{-}$ could therefore be decreased at the site of entrance into the xylem via competition for the same channel (Rubinigg et al., 2003). Moreover, the decrease of nitrogen could also be due to a reduction in nitrate influx resulting from an interference of external chloride with one or more types of transporters at the root plasma. Accumulation of chloride in leaf tissue at the highest Cl^-^:${\mathrm{NO}}_{3}^{-}$ ratio did not reduce the total leaf biomass, confirming that cardoon can be considered as a salt-tolerant crop (Ksouri et al., 2012; Pace et al., 2012).

It has been generally accepted that the critical toxicity concentration of chloride in leaf tissues ranges from 4 to 7 and from 15 to 50 g kg^-1^ on dry weight basis for Cl-sensitive and Cl-tolerant species, respectively (Marschner, 2012). In the present work chloride concentration in cardoon leaves recorded in the 20:80 ${\mathrm{NO}}_{3}^{-}$:Cl^-^ treatment was below the critical threshold (e.g., 50 g kg^-1^ DW; **Table 2**); this could explain the lack of biomass reduction recorded under high chloride concentration in the nutrient solution.

High chloride concentration in the nutrient solution interferes with several aspects of plant physiology including pigment biosynthesis and the activity of the photosystem (PS) II (Colla et al., 2010). Since the response of ${\mathrm{NO}}_{3}^{-}$:Cl^-^ ratio depends on the cardoon ability to counteract the effects induced by the four nitrate to chloride ratios, the lack of changes between treatments on the leaf dry biomass should be also reflected at physiological level. This was indeed the case, since no significant difference between the four nitrate to chloride ratios was observed for chlorophyll content (SPAD index) and the maximum quantum efficiency of PSII (F_v_/F_m_), frequently used as an indicator of the photoinhibition or stress damage to the PSII (Calatayud and Barreno, 2004; Colla et al., 2013b).

Nutraceutical and pharmaceutical properties of cardoon leaves are linked to the high concentration of antioxidant molecules in particular phenolic acids and flavonoids, as well as their high antioxidant activity (Rouphael et al., 2012a; Colla et al., 2013a; Borgognone et al., 2014). Moreover, the concentration of bioactive compounds (e.g., flavonoids and phenolic acids) varies significantly in relation to genetic, environmental, agronomic, and biotic/abiotic factors (Pandino et al., 2010; Rouphael et al., 2012b). Under stress conditions plants activate a series of counteracting measures including physiological and molecular mechanisms that consent acclimation to a sub-optimal environment (Atkinson and Urwin, 2012). Among these measures, the accumulation of specific molecules and secondary metabolites with multiple functions has a crucial role of ensuring plant growth under unfavorable conditions. These antioxidant molecules are thought to contribute in ROS scavenging and cellular water homeostasis (Bandyopadhyay et al., 2012; Sharma et al., 2012). More interestingly, the highest phenolics and flavonoids concentration recorded under high chloride concentration (**Table 3**) are also important to human health, and therefore they attribute an *added value* (compared to the control) to the nutraceutical properties of cardoon leaf extract. These results are very important because several pharmacological studies have demonstrated that cardoon extracts are supposed to exert antimicrobial, anti-inflammatory, anti-oxidative, anti-carcinogenic, bile-expelling and urinative activities, as well as the ability to inhibit low-density lipoprotein (LDL) oxidation and cholesterol biosynthesis (Falleh et al., 2008; Kukíc et al., 2008; Kammoun et al., 2010; Mileo et al., 2012).

Horticultural crops are usually supplied with high concentrations of inorganic nutrients, particularly nitrates which are the major anions responsible for biomass accumulation (Le Bot et al., 2001). Although this practice prevents the growth from being limited by mineral supply, it can lead to excessive plant vigor, decreasing quality, causing delay in harvest, nitrate accumulation in leaf tissues and high environmental impact (Stewart et al., 2001; Chen et al., 2004; Becker et al., 2015). An effective and sustainable fertigation strategy to limit nitrate waste in the environment while maintaining yield could be to terminate nitrate supply in the nutrient solution several days prior to final harvest. Nitrate withdrawal in the solution few DBH has been successfully used for lowering nitrate content in leafy vegetables with or without affecting crop productivity (Santamaria et al., 1998; Gonnella et al., 2004; Gent, 2012). In fact, the results of Experiment 2 clearly demonstrated that it is possible to withdraw nitrates in the nutrient solution from 5 up to 15 DBH without any negative effects on leaf dry biomass. Therefore, the standard nutrient recipes normally used in floating raft culture appear to be luxurious (Le Bot et al., 2001). Our results are in line with those of Santamaria et al. (1998) and Gonnella et al. (2004), who observed that reducing and/or eliminating nitrates in the nutrient solution few days (2–5) before harvesting can significantly reduced leaf nitrate concentration without affecting yield of chicory, rocket and lamb’s lettuce. ‘This acclimation appears the result of upregulation of nutrient uptake by plant roots as well as increasing in root biomass’ (Siddiqi et al., 1998) and may also depend on the plant species. Moreover, nitrogen limitation can negatively affect photosynthesis by decreasing available chlorophyll and disturbing photosynthesis owing to starch accumulation (Stewart et al., 2001). This was partially observed in the Experiment 2 since the chlorophyll content was not influenced by nitrate deprivation at 59 and 97 DAS, but was only affected at 124 DAS (**Table 4**).

The decrease in leaf nitrate content observed under N-deprivation was more pronounced than the reduction in total nitrogen (**Table 5**). Thus, up to 15 days of nitrate withdrawal could lower nitrate without sharply reducing total nitrogen or affecting growth and biomass of cultivated cardoon. These results are consistent with the findings of Gent (2012) who demonstrated that tissue nitrate changed more rapidly than total N over a 6-day interval of nitrogen depletion without affecting yield of hydroponic lettuce. The lowest nitrate concentration in cultivated cardoon leaves after 15 days of N-deprivation would suggest that a significant part of nitrate was assimilated in the ‘metabolic pool’ since the external ${\mathrm{NO}}_{3}^{-}$ is exhausted, while little would have remained available to be stored in cell vacuoles or ‘reserve pool’ as osmotic regulator (Santamaria et al., 1998). Contrarily, in the control treatment, the highest nitrate concentration in leaf tissue indicates that a greater part of nitrate was stored in the ‘reserve pool’ (Izmailov et al., 1992).

In the present study, it was also found that nitrate application can be eliminated 10–15 DBH with a significant increase in cardoon leaf quality as expressed by total phenols, ferric-reducing antioxidant power (FRAP) and total flavonoids (**Table 6**). It has been demonstrated that nutrient deficiency resulted in enhancing phenolic compounds in different leafy vegetables due to upregulation of the phenylpropanoid pathway, whose key enzyme, phenylalanine ammonia-lyase (PAL) is the link between primary and secondary metabolism (Oh et al., 2009; Becker et al., 2015). In a recent review, Stefanelli et al. (2010) elucidated how high nitrogen availability could inhibit phenolic production and subsequently antioxidant capacity, in several leafy vegetables. In line with our findings, highest total plant phenolics (TPP) in Chinese cabbage were observed in plants grown in soil without N fertilization while higher N application rates resulted in lower TPP and antioxidant activity (Zhu et al., 2009). Moreover, highest phenolic content in basil, and leaf lettuce grown hydroponically was also found after minimal nitrogen application (Nguyen and Niemeyer, 2008; Becker et al., 2015). Similarly to total phenols, the total flavonoids in leaf tissue increased linearly after 5, 10, or 15 days of nitrate depletion (**Table 6**). Increased flavonoids accumulation under low N availability has been reported previously in basil, broccoli and tomato leaves (Stewart et al., 2001; Jones et al., 2007; Nguyen and Niemeyer, 2008). An explanation for increased flavonoids synthesis under nitrogen stress is the possible activation of the flavonoids pathway through a number of enzymes and genes (Bongue-Bartelsman and Phillips, 1995) but does not have a pronounced influence on the shikimate pathway which produce phenylalanine (Lillo et al., 2008). It could be also expected that N deficiency (i.e., deprivation) could increase concentrations of flavonoids as they could be involved in the ROS (i.e., hydrogen peroxide) scavenging cascade (Takahama and Oniki, 1997).

## Conclusion

The results of Experiments 1 and 2 showed that floating raft culture appears to be a promising tool to obtain valuable cardoon production as well as improving quality aspects of leaf through proper management of nitrates in the nutrient solution. The use of nutrient solution having a ${\mathrm{NO}}_{3}^{-}$:Cl^-^ ratio of 20:80 could be adopted in the present conditions to improve the quality aspects of cardoon leaves by increasing antioxidant activity, total phenols and flavonoids and by lowering the nitrate concentration, with no negative impact on yield. The results also indicated that it is possible to withdraw nitrates from the nutrient solution 5, 10 and even 15 DBH without having a detrimental effect on yield. In addition, this will increase leaf quality by increasing total phenols, flavonoids and antioxidant capacity, especially when nitrate deprivation occurred for 15 DBH. Finally, it must be pointed out that nitrate withdrawn strategy may be valid provided that enough nitrates are accumulated in the plant tissues during the first part of the growing cycle.

## Author Contributions

DB conducted the trial as part of her Ph.D. program. YR was involved in the agronomic measurements and interpretation of the results. MC conducted the mineral analysis and interpretation of the results. LL made the analysis of total phenolics and flavonoids. GC set up the research program and he coordinated the research group in conducting the trial, statistical analysis, interpretation of results. All authors were involved in writing the manuscript and statistical analysis.

## Conflict of Interest Statement

The authors declare that the research was conducted in the absence of any commercial or financial relationships that could be construed as a potential conflict of interest. The reviewer RP declared a shared affiliation, though no other collaboration, with one of the authors YR to the handling Editor, who ensured that the process nevertheless met the standards of a fair and objective review.
